# Successful hematopoietic stem cell transplantation for two patients with relapse of intrachromosomal amplification of chromosome 21-positive B-cell precursor acute lymphoblastic leukemia

**DOI:** 10.3389/fped.2022.960126

**Published:** 2022-09-08

**Authors:** Tomoya Harada, Hidemi Toyoda, Naoki Tsuboya, Ryo Hanaki, Keishiro Amano, Masahiro Hirayama

**Affiliations:** Department of Pediatrics, Mie University Graduate School of Medicine, Tsu, Japan

**Keywords:** relapsed acute lymphoblastic leukemia, iAMP21, hematopoietic stem cell transplantation, late bone marrow relapse, minimal residual disease, B-cell precursor acute lymphoblastic leukemia

## Abstract

In children with relapsed acute lymphoblastic leukemia (ALL), it is essential to identify patients in need of treatment intensification. Minimal residual disease (MRD)-based treatment stratification resulted in excellent survival in children with late relapsed B-cell precursor (BCP)-ALL. Chemotherapy alone produced a favorable outcome in patients with negative MRD after induction. The genetic abnormality also plays an important role in determining the prognosis and stratification for treatment. Intrachromosomal amplification of chromosome 21 (iAMP21) is associated with a poor outcome and a high risk for relapse, and there is no standard treatment after relapse. Herein, we present two patients with relapsed iAMP21-positive ALL who were successfully treated by cord blood transplantation (CBT). Although both patients had late bone marrow relapse and favorable MRD response, CBT was performed due to iAMP21 positive. Patients 1 and 2 have been in remission post-CBT for 15 and 45 months, respectively. Patients with relapsed iAMP21-positive ALL may be considered for stem cell transplantation even in late relapses and favorable MRD response.

## Introduction

Childhood B-cell precursor acute lymphoblastic leukemia (BCP-ALL) is highly curable, but relapse is the main reason for treatment failure (1, 2). After relapse, the duration of clinical remission from the first diagnosis has been shown to be the strongest predictor of survival, with patients relapsing on or shortly after stopping chemotherapy having the worst outcome (1, 3). For late relapse, minimal residual disease (MRD) levels at the end of induction therapy are predictive of survival. Although allogeneic hematopoietic stem cell transplantation (HSCT) is recommended for patients with a higher MRD level post-induction, those with a low MRD level are allocated to not undergo HSCT and to continue chemotherapy (4, 5).

In pediatric ALL, chromosomal abnormalities are routinely used in risk stratification for treatment, which has substantially contributed to the remarkably improved outcomes of 90% survival for childhood BCP-ALL (2). Intrachromosomal amplification of chromosome 21 (iAMP21) is a recently identified chromosomal abnormality in childhood BCP-ALL, with an incidence of approximately 2% in older children with BCP-ALL (6–11). iAMP21 is detected during routine screening for the presence of the *ETV6-RUNX1* fusion by fluorescence *in situ* hybridization (FISH), and the finding of ≥ 3 extra *RUNX1* copies on a single abnormal chromosome 21 (a total of ≥ 5 *RUNX1* signals per cell) is currently used to define iAMP21 (8).

Patients newly diagnosed with iAMP21-positive BCP-ALL had poor survival when receiving standard therapy, but benefited from more intensive chemotherapy as a high-risk group (6, 9, 10, 12, 13). However, a standard treatment strategy for relapsed iAMP21-positive BCP-ALL is unknown. Herein, we describe two patients with relapsed iAMP21-positive BCP-ALL who received chemotherapy followed by cord blood transplantation (CBT).

## Results

### Case 1

**FIGURE 1:**
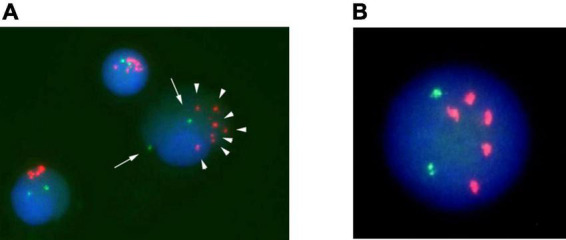
Fluorescence *in situ* hybridization (FISH) analysis of Case 1 on interphase with an LSI *ETV6/RUNX1* ES dual color probe. *RUNX1* signals are in red, and *ETV6* signals are green. **(A)** Cells at disease onset. **(B)** Cell at relapse.

A 9-year-old boy was admitted due to fever for two weeks and lumbago. The laboratory results showed a white blood cell (WBC) count of 3.9 × 10^9^/L with 0% blast cells, and bone marrow (BM) evaluation showed 50% blast cells with lymphoid characteristics. Immunophenotyping confirmed BCP-ALL. Cytogenetic analysis was performed on a BM sample. G-banded chromosome analysis revealed a male karyotype 46, XY, -21, + mar/46, XY. The poor quality of the metaphase chromosomes restricted their evaluation. The FISH study showed no rearrangements in *BCR/ABL1*, *KMT2A*, or *ETV6/RUNX1*. However, FISH revealed 5–12 signals of *RUNX1* in 68% of interphase cells (Figure 1A). There was no extramedullary organ infiltration. He was treated according to the Japanese Pediatric Leukemia/Lymphoma Study Group’s protocol for the standard-risk group (14). Peripheral blood showed good treatment response to prednisolone on day 8, and BM examination after the induction therapy revealed complete remission (CR) and negative for MRD detected by a real-time polymerase chain reaction of antigen receptors (15). Although he had been clinically well for 35 months, he presented pallor and splenomegaly at 11 months after frontline chemotherapy. He had a WBC count of 15.9 × 10^9^/L with 66% blast cells and the morphologic evaluation of the BM showed 97.3% lymphoid blast cells. G-banded chromosome analysis of the BM sample revealed a male karyotype 46, XY, -21, + mar. FISH analysis revealed no *ETV/RUNX1* fusion signals and five signals for the *RUNX1* probe in 96% of the analyzed nuclei (Figure 1B). There was no extramedullary organ infiltration. He was diagnosed with late isolated BM relapse of iAMP21-positive BCP-ALL and treated according to the ALL-R3 mitoxantrone regimen (16). He achieved a second CR after induction chemotherapy and MRD in BM after induction was negative. He received two cycles of a 28-day continuous infusion of blinatumomab at 15 μg/m^2^/day, followed by a 4/6 antigen matched unrelated CBT. The myeloablative conditioning regimen consisted of 12-Gy total body irradiation (TBI) from days -9 to -6 (six fractions), intravenous etoposide 15 mg/kg/day from days -5 to -4, and intravenous cyclophosphamide 60 mg/kg/day from days -3 to -2. Tacrolimus and short-term methotrexate were used for graft-versus-host disease (GVHD) prophylaxis. Neutrophil engrafted on day 23 post-CBT. BM examination post-CBT revealed CR with 100% donor-type hematopoiesis. He experienced grade II acute GVHD involving the skin only, which was successfully treated with an increased tacrolimus dose. He has been in good condition for the last 15 months following CBT without ALL recurrence.

### Case 2

**FIGURE 2:**
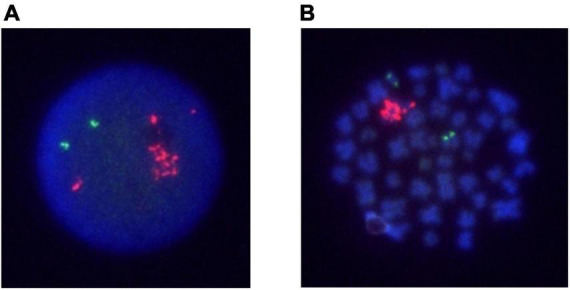
FISH analysis of Case 2 on interphase and metaphase with an LSI *ETV6/RUNX1* ES dual color probe. *RUNX1* signals are in red, and *ETV6* signals are green. **(A)** An interphase cell at relapse. **(B)** A metaphase cell at relapse.

A 15-year-old girl was admitted to the hospital because of petechiae and was diagnosed with BCP-ALL. At the onset of the ALL, the BM sample revealed a female karyotype 46, XX, and FISH analysis for *ETV/RUNX1* was not performed. Chemotherapy was started according to the Japan Adult Leukemia Study Group B-ALL213 protocol (17, 18). The patient achieved CR after induction chemotherapy. Although she had been clinically well for 36 months, leukocytopenia was detected during regular consultation 12 months after frontline chemotherapy. A diagnostic test revealed a WBC of 2.8 × 10^9^/L and 90% lymphoid blast cells in the BM. G-banded chromosome analysis of the BM sample revealed a female karyotype 46, XX, and FISH analysis revealed no *ETV/RUNX1* fusion signals and 5–10 signals for the *RUNX1* probe in 83% of the analyzed nuclei (Figure 2). Central nervous system infiltration was not found. She was diagnosed with late isolated BM relapse of iAMP21-positive BCP-ALL and treated according to the ALL-REZ BFM 2002 regimen (19). MRD in BM after induction was negative. On week 19 of chemotherapy, she received a 4/6 antigen matched unrelated CBT. The myeloablative conditioning regimen consisted of 12-Gy TBI from days -8 to -5 (six fractions), intravenous etoposide 1800 mg/m^2^ at day -4, and intravenous cyclophosphamide 60 mg/kg/day from days -3 to -2. Tacrolimus and short-term methotrexate were used for GVHD prophylaxis. Neutrophil engrafted on day 19 post-CBT. BM examination post-CBT revealed CR with 100% donor-type hematopoiesis. Grade I acute GVHD involving the skin only was well controlled by adjustment of tacrolimus. She has been in good condition for the last 45 months following CBT.

## Discussion

We present two late BM relapsed cases of iAMP21-positive BCP-ALL that were successfully treated by CBT. The duration of the first CR and immunophenotypes predict outcomes in patients with relapsed ALL (20). Patients with late BM relapses (BCP-ALL relapses occurring > 6 months after stopping chemotherapy) have relatively favorable survival rates (16, 19). MRD is a highly predictive risk factor in late BM relapses of BCP-ALL and is successfully used for treatment stratification in relapse protocols for childhood ALL (16, 19). Patients with high and low MRD after induction therapy were allocated to undergo allogeneic hematopoietic stem cell transplantation (HSCT) and to receive chemotherapy, respectively (16, 19). Even though both patients had late BM relapses with low MRD after induction, they underwent CBT because of iAMP21 positivity.

In patients with newly diagnosed iAMP21-positive BCP-ALL, the presence of iAMP21 is associated with an increased risk of relapse, and the patients benefited from more intensive frontline therapy as a high-risk group (9, 10, 12). However, a standard treatment strategy for relapsed iAMP21-positive BCP-ALL is unknown. Relapsed iAMP21-positive BCP-ALL was categorized as high-risk cytogenetic groups in the ALL-R3 trial and ALL-REZ BFM 2002 trials (16, 20). In long-term follow-up of late BM relapses in the ALL-R3 trial, progression-free and overall survivals were poor in cytogenetic high-risk groups such as iAMP21, KMT2A, TCF3-PBX1, low hypodiploid (16).

Patients with high-risk relapses (BCP-ALL relapses within 18 months of the first diagnosis or with isolated medullary relapses within 6 months of stopping therapy) have poor event-free survival rates (22.6%) (20). Maintaining a second CR remains a major problem in high-risk BCP-ALL relapses. The second CR after HSCT was maintained in 61 (47%) of 131 patients with a high-risk BCP-ALL relapse (20), showing HSCT benefits survival even in high-risk cases. Thus, relapsed iAMP21 should be treated as a high-risk relapse and might require HSCT even in late relapse and negative MRD cases after induction.

The use of pediatrics-inspired protocols in adolescent and young adults ALL results in superior survival compared with the adult protocols (21). Although adolescents and young adults with ALL are currently treated with pediatric protocols in Japan, case 2 did not have the opportunity to be treated with pediatric protocols at the time of disease onset. Since this might be one of the reasons for relapse, adolescents and young adults with ALL should be treated with pediatric protocols. iAMP21 patients have been shown to clearly benefit from receiving more intensive therapy (9, 10, 12). However, iAMP21 is not assigned to a more intensive regimen in the current Japanese pediatric protocol for newly diagnosed BCP-ALL. Heerema et al. reported that MRD and iAMP21 were independently prognostic in newly diagnosed standard-risk ALL (12). In those trials, standard-risk patients who were MRD negative and iAMP21 positive had worse EFS than standard-risk MRD-negative patients without iAMP21 (4-year EFS, 82.2% ± 9.0% v 94.2% ± 0.5%, respectively; *P* < 0.001) (12). Moreover, late complications such as secondary cancers and organ-specific complications after HSCT substantially contribute to long-term morbidity and mortality (22). Therefore, to avoid disease relapse and late HSCT effects, newly diagnosed iAMP21 cases should receive intensive frontline chemotherapy as a high-risk group, even if they had good MRD response at end induction.

In order to know whether there were other iAMP21 positive patients with BCP-ALL, the medical records of patients diagnosed with BCP-ALL between 2012 and 2021 in Mie University Hospital were retrospectively reviewed. Although FISH images for *ETV6/RUNX1* performed in 42 patients were evaluated, other iAMP21 positive patients were not identified. A nationwide survey is necessary to determine the incidence and prognosis of iAMP21 in patients with BCP-ALL in Japan.

In conclusion, patients with iAMP21-positive BCP-ALL relapse could be successfully rescued by HSCT. Further clinical trials are needed to determine the appropriate treatment for patients with iAMP21-positive BCP-ALL relapse.

## Data availability statement

The raw data supporting the conclusions of this article will be made available by the authors, without undue reservation.

## Ethics statement

Written informed consent was obtained from the individual(s), and minor(s)’ legal guardian/next of kin, for the publication of any potentially identifiable images or data included in this article.

## Author contributions

TH participated in data collection, analyzed, interpreted the data, performed the analysis, and wrote the manuscript. HT conceived and designed the analysis, supervised data collection, analyzed, interpreted the data, and wrote the manuscript. NT, RH, and KA participated in data collection, analyzed, and interpreted the data. MH conceptualized the study, supervised data collection, and reviewed the manuscript. All authors approved the final manuscript as submitted and agreed to be accountable for all aspects of the work.

## Abbreviations

ALL: Acute lymphoblastic leukemia
BM: Bone marrow
BCP-ALL: B-cell precursor acute lymphoblastic leukemia
CBT: Cord blood transplantation
CR: Complete remission
FISH: Fluorescence *in situ* hybridization
GVHD: Graft-versus-host disease
HSCT: Hematopoietic stem cell transplantation
iAMP21: Intrachromosomal amplification of chromosome 21
MRD: Minimal residual disease
TBI: Total body irradiation
WBC: White blood cell.
